# Brief, effective experience to increase first-year medical students’ nutrition awareness

**DOI:** 10.1080/10872981.2021.1896160

**Published:** 2021-03-11

**Authors:** Mary Thoesen Coleman, Paula Rhode Brantley, Pamela Markiewicz Wiseman, M. Robin English, Lauri Byerley

**Affiliations:** a Department of Family Medicine, School of Medicine, Louisiana State University Health Sciences Center New Orleans, New Orleans, LA, USA; b Office of Undergraduate Medical Education, School of Medicine, Louisiana State University Health Sciences Center New Orleans, New Orleans, LA, USA; c Department of Physiology, School of Medicine, Louisiana State University Health Sciences Center New Orleans, New Orleans, LA, USA

**Keywords:** Nutrition awareness, nutrition education, first-year medical students, nutrition training, nutrition wellness

## Abstract

**Background**: Wellness is an important concept for medical students to learn, both for their own health and for their patients. Since nutrition is an essential part of one’s wellness that can positively or negatively impact one’s health, it is important for medical students to learn approaches to nutritional wellness. Studies have shown that physicians’ nutrition attitudes and clinical practices are positively correlated with their dietary practices.

**Objective**: Here, we describe a brief nutrition-based education experience for first-year students offered at the start of the medical school curriculum that is designed to increase their nutrition awareness.

**Design**: The nutrition experience involved five components: 1) having students complete three 24-hour food recalls; 2) comparing their recalls to nutrient standards; 3) emphasizing strategies that include simple, nutritionally sound food choices and preparation; 4) surveying students on their implementation of personal healthy nutritional strategies; and 5) requesting future recommendations for modifying the educational experience.

**Results**: Most students’ diets did not meet the recommended dietary levels for several nutrients, and these deficiencies corresponded to specific food group inadequacies. Forty percent of the students responded to a three-month follow-up survey. Of these students, 46% implemented one of the presented strategies to improve their food intake. Most changes included the addition or deletion of a particular food. Seventy-three percent recommended repeating the program in the future.

**Conclusions**: We demonstrate that a brief 2.5-hour nutrition wellness experience can increase nutrition awareness and promote dietary change in incoming medical students. Many felt that the experience was valuable and recommended offering a similar experience to future classes.

## Introduction

Attention to wellness is increasingly recognized as an important component of medical education, particularly since burnout has recently been identified as a significant problem for medical students [1]. One response to the increased awareness of medical student burnout has been the creation of wellness programs, many of which include a nutrition component.

The idea of adding wellness programs to medical curricula as a mechanism to improve students’ health is not new. Vanderbilt’s program has an active student leadership component that is largely extracurricular. It includes online exercise routines, yoga, tai chi, meditation, study breaks, festival events, optimized student lounge and study space, and increased availability of health information sessions. Cooking classes for healthful food options are also offered [2]. Stanford has incorporated wellness into its preclinical as well as clinical curriculum by offering a variety of wellness electives, including a lunchtime elective on popular and clinical nutrition topics [3]. Dartmouth has integrated evidence-based nutrition content across all four years of its curriculum [4].

Medical students have reported healthier lifestyle habits than their peers upon entering medical school [5,6], but maintaining those healthy behaviors during medical school is challenging [7]. Studies have suggested a decline in physical activity, diet quality, life satisfaction, and general health during medical school [8–10]. This decline can translate into less frequent nutrition counseling by physicians because they do not feel confident advocating for something they do not practice [11].

Since there is little doubt that nutrition knowledge and dietary habits affect the wellness of both medical students and their future patients, it is important that medical students become acquainted with the effect nutrition can have on their health and that of their patients. Although medical schools are increasingly addressing nutrition, including it either as a component of a wellness program or integrated into the curriculum, few US medical schools adequately address nutrition. Adams et al. reported that most US medical schools fail to provide the recommended minimum of 25 hours of nutrition education, and 36% provide less than half that much [12]. Several studies have shown that improving a physician’s nutrition counseling skills could reduce the incidence of obesity, hyperlipidemia, and diabetes [13,14].

Medical students, like most individuals, have gaps in their nutrition knowledge and dietary habits, which, if addressed, could help with handling any potential adverse effects of a high-stress environment. Diets such as the Okinawan diet, Mediterranean diet, and DASH (Dietary Approaches to Stop Hypertension) diet share features such as being high in vegetables and fruit but reduced in meat, refined grains, saturated fat, sugar, and salt. This balance is thought to increase antioxidant intake and lower glycemic load, thus contributing to a healthier lifespan [15,16]. Certain foods, particularly those incorporating complex carbohydrates, proteins, vitamin C, B vitamins, magnesium, and selenium, are thought to play an important role in stress management [17]. In a 2016 analysis of the dietary practices of pharmacy and medical students at ten schools in California, only 50% had a saturated fat intake of <10% of total kcal, 13% met their fiber intake goals, and 10% consumed greater than eight servings/day of fruit and vegetables [18]. Fifty-nine percent cited lack of time as the biggest barrier to a healthful diet.

In order to respond to these challenges, we describe a brief, novel, nutrition-based educational experience for first-year students that was integrated early into a concentrated medical school curriculum and designed to increase each student’s nutrition awareness.

## Materials and Methods

An interactive nutrition experience was developed as part of a more extensive wellness program, which included instruction on sleep hygiene, study habits and time management skills, general self-care, exercise, and mindfulness, for first-year medical students. Each component was designed to make the students mindful of nutrition’s importance in helping them succeed during medical school. Table 1 describes the components, objectives, strategies, and outcomes used in designing the nutrition experience while keeping in mind the limited time available in the medical school curriculum. The students spent, on average, a total of 2.5 hours to complete this experience.

**Table 1.** The components, objectives, strategies, and outcomes utilized to increase student’s nutrition awareness

Component		Objective	Strategy	Outcome
Food recall	Having students complete three 24-hour food recalls	Complete recall of food intake	Student completion of ASA 24-hour recall	Nutrient and food group analysis of three 24-hour food recalls
Educational Session – Food recall review	Comparing student food recalls to nutrient standard	Determine strengths and weaknesses in individual student’s and group’s dietary intake	Sharing of nutrient analysis for both individual and group recalls and comparison to nutrient standards	Nutrients or food groups identified in personal diets that met or exceeded the recommended intake Student individual intake compared to class aggregate intake Class intake compared to standard dietary recommendations
Educational Session – MyPlate	Emphasizing strategies that include simple, nutritionally sound food choices and preparation	Provide simple educational strategies that include nutritionally sound food choices and food preparation applicable for both personal and professional use	Faculty PowerPoint presentation delineating MyPlate dietary plan	Introduction to My Plate, a healthy dietary eating plan based on the appropriate intake of key food groups
Educational Session – Interactive discussion	Emphasizing strategies that include simple, nutritionally sound food choices and preparation	Provide simple educational strategies that include nutritionally sound food choices and food preparation applicable for both personal and professional use	Interactive group discussion between class and faculty regarding tips for healthy eating and food preparation using visual aids	Exchange among faculty and students, sharing their own healthy tips for ‘what works.’
Survey -Assessing dietary changes	Surveying students on their implementation of personal healthy nutritional strategies	Identify and assess changes made to diet based on nutrition component	Post-learning experience survey of students on implementation of personal healthy nutritional strategies	Student report of successfully implemented dietary changes Student report of barriers to dietary changes
Survey – Evaluation and recommendation	Requesting future recommendations for modifying the educational experience	Establish the value of a brief nutrition-based wellness component in the curriculum on implementing healthy dietary changes Assess and improve the educational quality of the brief nutrition-based wellness component in the curriculum	Post-educational survey requesting evaluation of and possible modifications for enhancing the educational experience	Student report of value provided by individual components of the brief nutrition-based wellness experience Student report of whether nutritional educational information was new Modifications of the educational learning strategy to optimize future learning and facilitation of healthy nutritional/dietary behaviors

The approach involved an active, hands-on learning component, a traditional PowerPoint presentation, an interactive large group discussion, and a three-month follow-up evaluation. Faculty from basic science (Department of Physiology), a clinical department (Family Medicine), and the Office of Undergraduate Medical Education collaborated to present this wellness-based nutrition experience. Attendance was mandatory for the food recall training (20 minutes) and educational session (1 hour). Completing the three 24-hour food recalls and end survey was not obligatory.

The Institutional Review Board at the Louisiana State University Health Sciences Center New Orleans reviewed this educational evaluation project. It determined that the activities described do not constitute human subjects research, and therefore IRB approval for this project was not required.

*Food Recall*: During the third week of medical school orientation, a Registered Dietitian (last author) instructed the students in a 20-minute session on how to keep an electronic log of their food intake using an automated, web-based, self-administered 24-hour dietary assessment (ASA24) (https://epi.grants.cancer.gov/asa24/) [19]. The ASA24 diet tracking program is freely available on the web and mobile devices and is funded and maintained by the National Cancer Institute. It enables multiple, automatically coded, self-administered 24-hour recalls and/or single or multi-day food diaries. The ASA24 provides values for 65 nutrients and 37 food groups; twenty-seven of the most common nutrients and five of the most common food groups are reported here (Table 2).

**Table 2.** Nutrient and food group intake of students

Nutrient	Daily Intake	Daily Value^a^	% of DV
Calories (kcal)	1868 ± 34^b^	2000	93.4
Protein (g)	95.1 ± 2	50	190.3
Fat (g)	77 ± 1.8	78	98.7
Total Carbohydrate (g)	199.2 ± 4.1	275	72.4
Dietary Fiber (g)	17.4 ± 0.4	28	62.1
Calcium (mg)	899.4 ± 22.8	1300	69.2
Iron (mg)	13.3 ± 0.3	18	74
Magnesium (mg)	308.7 ± 6.4	420	73.5
Phosphorus (mg)	1455.2 ± 28.8	1250	116.4
Potassium (mg)	2589.7 ± 53.6	4700	55.1
Sodium (mg)	3549 ± 72.2	2300	154.3
Zinc (mg)	11.1 ± 0.3	11	100.5
Selenium (mcg)	131.7 ± 2.9	55	239.5
Vitamin C (mg)	74.3 ± 3.3	90	82.6
Thiamin (mg)	1.5 ± 0	1.2	129.1
Riboflavin (mg)	2.1 ± 0	1.3	158
Niacin (mg NE [1])	27.5 ± 0.7	16	172
Vitamin B6 (mg)	2.4 ± 0.1	1.7	141.1
Folate/Folic Acid (mcg DFE)	394.8 ± 9.6	400	98.7
Vitamin B12 (mcg)	5.4 ± 0.4	2.4	225.4
Vitamin A (mcg RAE [1])	740.2 ± 39.5	900	82.2
Vitamin K (mcg)	212 ± 12.8	120	176.7
Cholesterol (mg)	349.6 ± 11.3	300	116.5
Saturated Fat (g)	23.9 ± 0.6	20	119.7
Vitamin D (mcg [1])	5.7 ± 0.4	20	28.3
Choline (mg)	365.8 ± 8.9	550	66.5
Vitamin E (mg alpha-tocopherol)	9.3 ± 0.3	15	62.3
Caffeine (mg/day)	97 ± 6		
Added sugars (g)	9.9 ± 0.4	50	19.8
Healthy Eating Index	59.2 + 0.9		
Kcal from Protein (%)	21.0 + 0.3	10–35% Kcal^c^	
Kcal from Fat (%)	36.5 + 0.4	20–35% Kcal	
Kcal from Carbohydrates (%)	42.7 + 0.5	45–65% Kcal	
Food Groups			
Fruits (cup)	0.9 ± 0.1	2	43.5
Vegetables (cups)	1.7 ± 0.1	2.5	68.6
Grains (ounces)	5.8 ± 0.2	6	96.5
Protein (ounces)	8 ± 0.2	5.5	145.3
Dairy (cups)	1.5 ± 0.1	3	50.8

^a^Daily value (DV) is a single value set by the Food and Drug Administration for specific nutrients and used on the nutrition facts panel found on packaged food labels. The DV is based on the recommended dietary allowance [49] and dietary recommended intake (DRI).

^b^mean ± SEM. Rows with gray shading are less than the recommended amount.

^c^US Dietary Guideline Recommendations

The students were instructed to complete three 24-hour recalls over a ten-day period. They were instructed to analyze two weekdays and one weekend day, and to print the results to view the analyses during the educational session. The students could send questions to the dietitian’s email address. The most frequent question was a request for directions on how to print the results.

*Educational Session*: After the students completed the food recall, the authors engaged the students in a one-hour educational session divided into three 20-minute discussions. A newly designed team-based learning room was chosen for this session. This room was selected because it accommodates the entire class, allows students to interact at tables in small groups, and facilitates faculty circulation around the room. Each group was provided a group computer, hookups for personal computers, and a group microphone for interaction with the entire class. Throughout the room, several large screens were used to project the PowerPoint slides and other interactive tools such as clicker questions. Students’ responses to personal diet questions such as ‘Did your diet contain enough calcium?’ were projected in real-time, then compared to the class aggregate data to emphasize individual and group similarities and differences.

During the first of three 20-minute discussions, the faculty provided students with results from the food recalls, including the group’s nutrient intake, food group intake, added sugar, and caffeine intake. Additional data demonstrating a connection between the suboptimal intake of food groups and deficiencies in nutrients emphasized the importance of particular food groups as a source of nutrients.

During the second 20 minutes, the faculty engaged the students in a discussion on healthy eating tips in the context of the US Department of Agriculture’s MyPlate Plan program (https://www.choosemyplate.gov/) [20], a dietary plan that stresses plant-based foods. MyPlate visually depicts a plate where half of the plate is vegetables and fruits, a quarter of the plate is filled with grains (about 50% whole grains), and a quarter is filled with protein along with a side serving of fat-free or low- fat dairy. The diet recommends avoiding processed foods and foods high in solid fats, added sugars, and salt.

Finally, during the last 20 minutes, a large group interactive discussion on tips for modifying behaviors to improve diet during medical school was led by three medical doctors (first, third, and fourth authors). Faculty and students shared ideas and tips for healthy eating during medical school, including examples of preparing healthy portable meals.

*Survey*: Three months later, students completed a survey about whether they had made any dietary changes based on the diet analysis and educational session they attended. A link to a Survey Monkey survey was sent by email. The survey consisted of ten questions with two parts: 1) assessing student’s dietary changes, and 2) evaluations and recommendations. There were three yes/no questions, three questions with branched Likert scale responses, and four questions with open-ended responses for optional comments.

*Statistical Analysis*: The dietitian downloaded ASA24 dietary information for all completed food recalls in a de-identified database and used it to provide feedback on the group’s aggregate dietary intake during the intervention experience. The group’s dietary data were analyzed using Excel (Office 365 ProPlus version), SAS version 9.4 (SAS Institute Inc, Cary, NC, USA), and GraphPad Prism version 9.0 for Windows (GraphPad Software, San Diego, CA USA, www.graphpad.com). The group’s average intake for each nutrient was compared to the Daily Value (DV) [21] and the food groups for a 2000 calorie diet [22]. DV and the MyPlate 2000 calorie diet rather than Dietary Recommended Intake (DRI) were used because the ASA24 data does not collect personal information such as sex, age, height, weight, or activity level required to determine the appropriate DRI level and to calculate individual energy requirements. DVs are the recommended amounts of nutrients to consume each day and are based on the DRI. The DV is found on the Nutrition Facts label on pre-packaged foods. The calorie level on the nutrition facts panel is 2000 calories per day [23], and DV for added sugars is no more than 50 g per day [24]. The food group data was compared to the standard 2000 kcal MyPlate Plan’s recommendations available on their website (https://www.myplate.gov/myplate-plan) [20]. The Healthy Eating Index [25] was calculated using the SAS code provided with the ASA24 Resources [26].

For each non-open-ended survey question, the frequencies and percentages for each response were calculated. For open-ended questions, each response was coded to one of five categories aligned with the educational components in Table 1: 1) food recall, 2) comparison to nutritional standards, 3) educational strategies, 4) post-intervention survey lessons, and 5) recommended changes to the experience. A single response could be assigned several codes, depending on the nature of the statement. The frequency of each code was determined, and a percentage was calculated. Within the food recall and strategy categories, the comments were further subcoded.

## Results

Two hundred and five students participated in the experience, of whom 198 students were newly admitted. Newly admitted students were an average age of 24 years (range: 21–40 years), 54% (106) female, and 46% (92) male. Self-reported race and ethnicity status were as follows: 69% (137) White; 7.5% (15)15 Black; 7.5% (15)(15) Hispanic and/or Latino/Spanish; 7.5% (15)15 Asian; 3.5% (7)7 Asian Indian; and 5% (10)10 Other.

One hundred and ninety-three students completed the food recall (94%). Fourteen completed one day of recalls (7%), 32 two days (17%), 138 three days (72%), and nine completed four days (4%). The values reported here and presented to the students during the educational session included all completed recalls regardless of the number of days each student completed. Each day was equally weighted. Students averaged 19 ± 0.5 minutes to complete one 24-hour food recall. The analysis of the food recalls included 27 nutrients, added sugar, and caffeine (Table 2). Eleven nutrients were consumed in a quantity that did not meet the Daily Value (DV): total carbohydrate, dietary fiber, calcium, iron, magnesium, potassium, vitamin C, vitamin A, vitamin D, choline, and vitamin E; fat and folate were slightly less than 100% (Table 2). These nutrient deficiencies were reflected in the student’s inadequate intake of the fruit, vegetable, grains, and dairy food groups. Consumption of the nutrient protein was almost two-fold higher than the daily amount needed. This increase was reflected by a higher intake of foods from the protein food group.

Sugar, caffeine, and alcohol are 3 non-nutrient substances of concern in the American diet. Notably, added sugar did not exceed the recommended 50 g amount. Likewise, the amount of caffeine consumed daily was similar to the amount found in one cup of coffee, and 177 students drank coffee at least once during the recall period. Thirty-five students (18%) reported alcohol intake on at least one day, either as a beverage or added to cooking; the ASA24 does not distinguish. Their average intake was 2.0 ± 2.7 (mean ± std dev) drinks/day.

**Figure 1.**
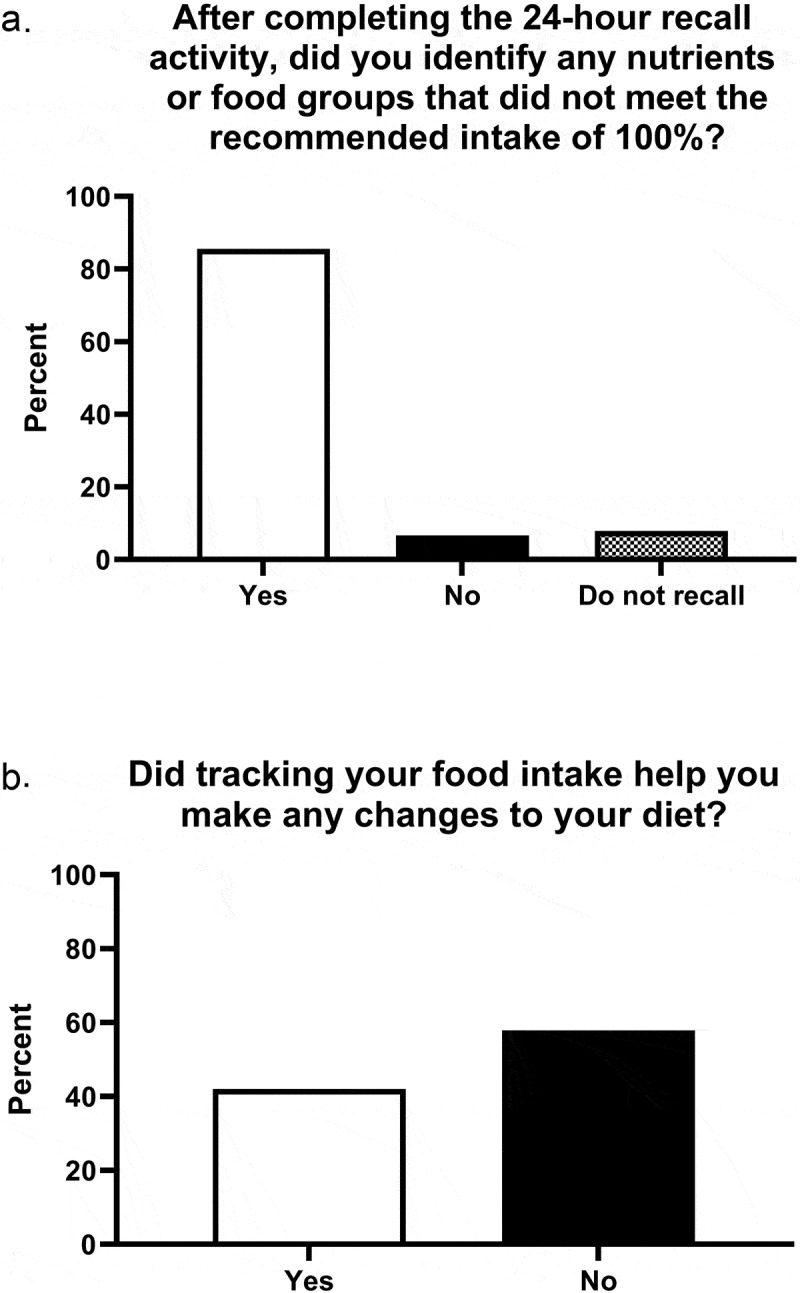
Impact of nutrient inadequacies on making dietary changes

**Figure 2.**
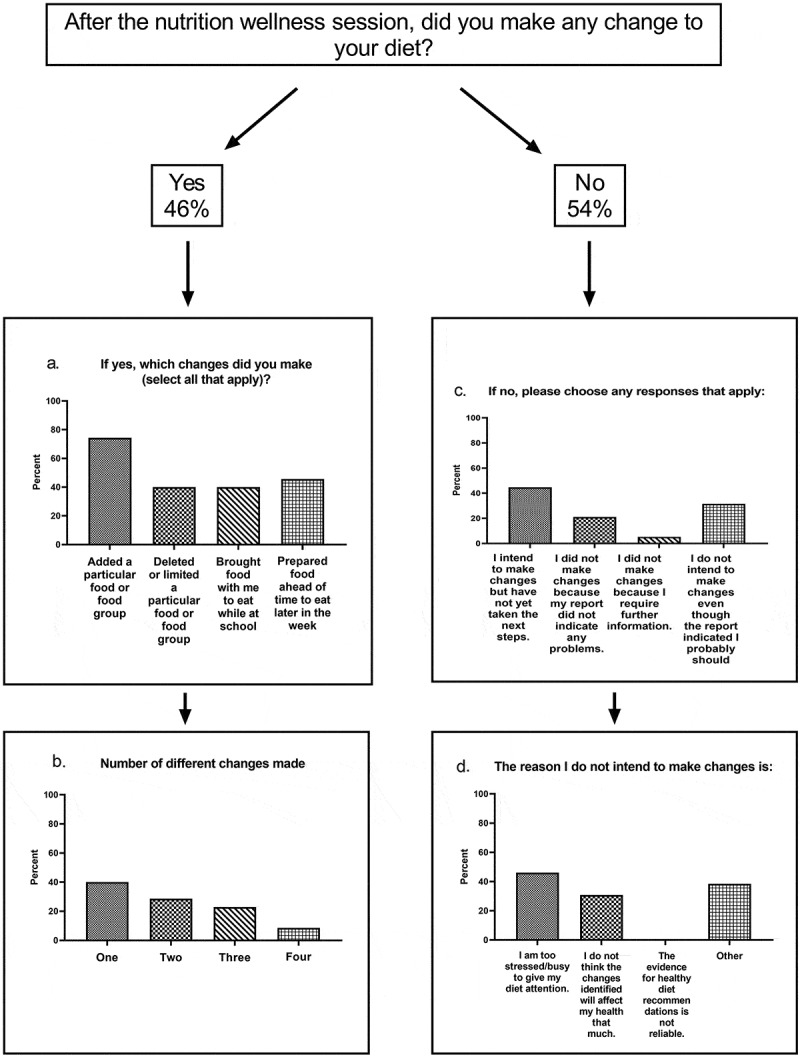
Impact of the nutrition wellness experience on making a dietary change

**Figure 3.**
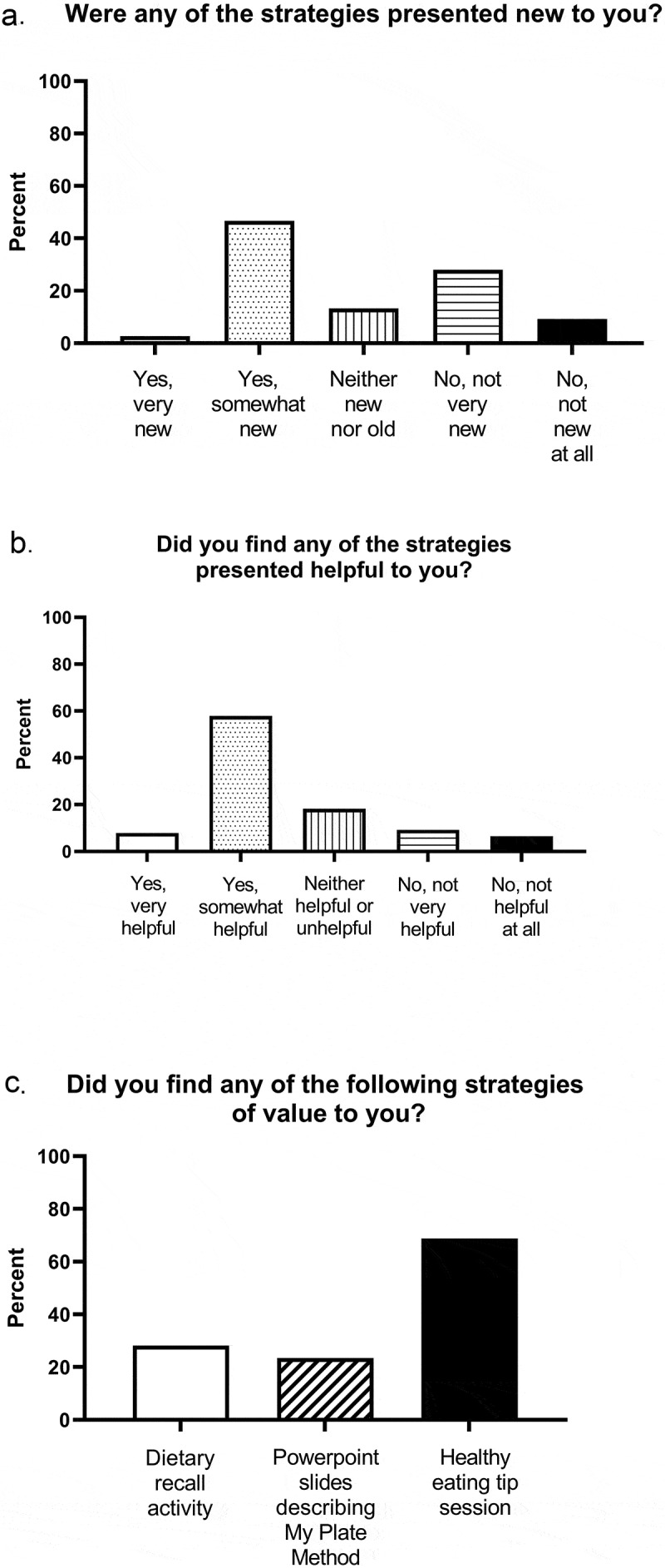
Novelty, helpfulness, and value of the strategies presented

**Figure 4.**
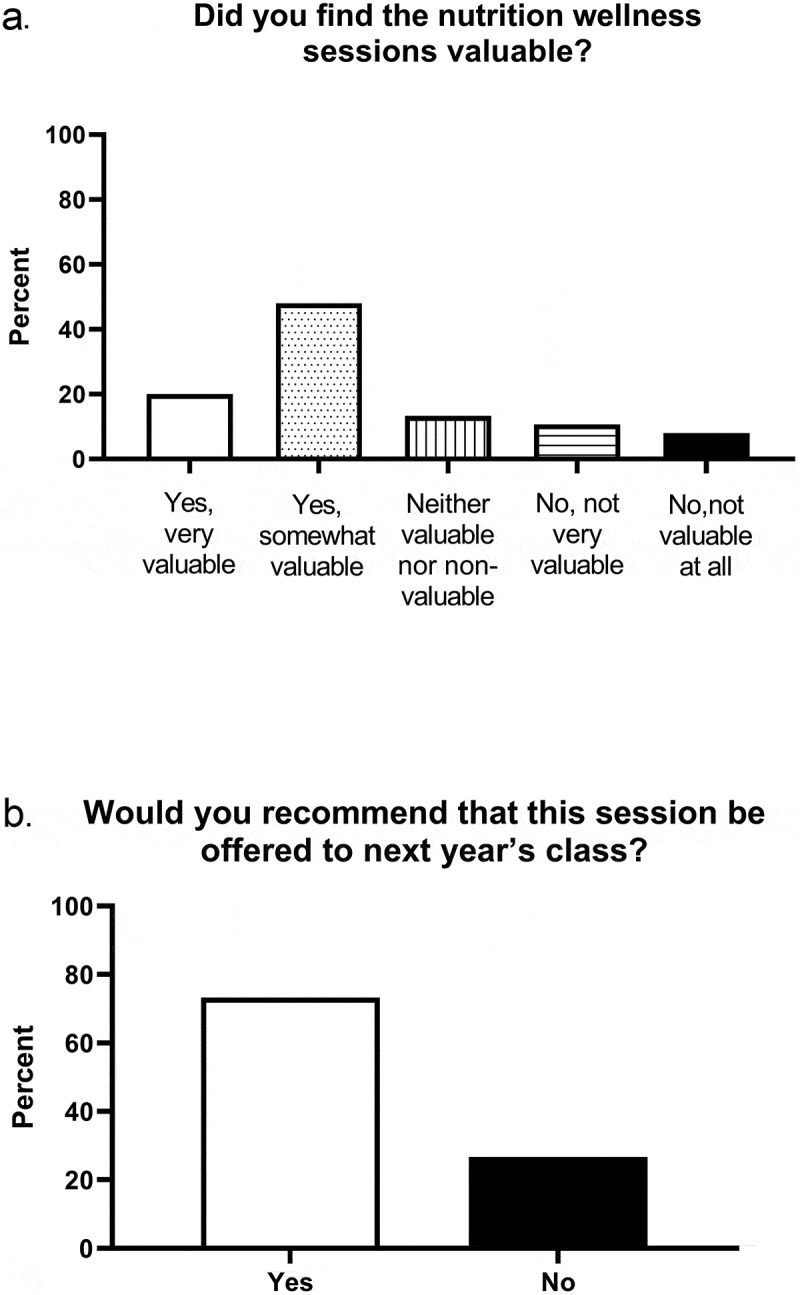
Value of the nutrition wellness experience and students’ recommendations

*Survey*: Forty percent (n = 78) completed the survey and 86% of these students reported their diet was lacking in at least one nutrient or food group (Figure 1A). Forty-two percent felt tracking their food intake helped them make a change to their diet (Figure 1B). Figure 1–4 reflect the wording, skip pattern, and flow of several of the survey’s questions, and the student response percentages of each.

Three months after the nutrition wellness experience, 46% of the students who completed the survey responded that they had implemented a change to their diet (Figure 2). For those students who changed their diet, the top strategy (74%) was adding a particular food or food group (Figure 2A). The foods that were added are not known. The group was evenly divided for the other strategies: deleting a particular food, bringing food with them to eat at school, and/or preparing food ahead. Interestingly, 40% used one strategy to implement change, while 60% used two or more strategies (Figure 2B).

Fifty-four percent of the students responding to the survey had not made a change at three months (Figure 2). Of these, 40% were planning to make a change but had not done so yet (Figure 2C). Thirty-two percent responded that they do not want to make a change even though their food recalls indicated that a change might be beneficial (Figure 2C). Thirteen respondents provided an explanation for not making the changes (Figure 2D). None felt the evidence was unreliable. Over half felt they were too stressed or busy to make the changes, while a third did not think the changes they needed to make would affect their health. Five provided other reasons; for example, they felt their diet was ‘pretty balanced,’ or they had already discovered foods that work best for them.

Approximately half of the survey respondents felt that at least some of the strategies presented were new (Figure 3A), and fifty-eight percent felt the strategies were helpful (Figure 3B). The tips for healthy eating, structured as an interactive shared discussion between faculty and students, were considered the most helpful (Figure 3C).

Overall, a majority of the survey respondents (68%) found value in the nutrition experience components of the overall wellness program, and 70% recommended that we offer the experience in the future (Figure 4). Twenty-nine of the students responded to the open-ended questions, ‘How would you improve the session? What suggestions do you have?’ Of these, eighteen percent commented that the food recall process was time-consuming, burdensome, cumbersome, or too repetitive. Forty percent suggested additional content such as eating on a budget, providing sustainable grocery shopping, hydration, and harms/benefits of excess protein intake. Thirty-five percent made suggestions about changing the future experience, for example, grading it and/or altering its timing within the curriculum.

## Discussion

This experience, designed to increase nutrition awareness, exposed medical students to their food habits and dietary deficiencies. For some students it promoted a change in their diet. The experience was placed early in the curriculum to emphasize the importance of nutrition for the students’ health. Several small studies have shown that nutrition training interventions can improve medical students’ dietary behaviors [25]. Also, physician’s dietary practices have been positively correlated with their clinical nutrition counseling attitudes [27] and practices [6,28,29], such that the healthier a doctor’s diet, the better they are at counseling their patients on a healthy diet. Other studies have shown that the incidence of diseases like obesity, hyperlipidemia, and diabetes could decline if physicians advised about nutrition [13,14]. Less than 50% of primary care physicians include nutrition or diet counseling in their patient visits [6,30–34]. The MyPlate framework provides a simple food group tool applicable for both personal and professional use in following a healthy diet.

We found that even a brief educational experience can have a meaningful impact on some students’ dietary behaviors. Ramsetty et al. [35] reported that student’s attitudes towards nutrition counseling changed after participation in a small group, online, video-conferencing discussion of two nutrition cases. The time commitment was estimated at one hour. In another study, dental students completed a three-week experiential exercise in which they changed one diet-related behavior, such as stopping the consumption of soft drinks. These fairly limited experiences were enough to positively impact the student’s behavior and increase their interest in helping patients change their diet-related practices [36]

Knowing that effective nutrition education can be brief is important because adding content to a busy medical school curriculum is difficult. Challenges include finding curricular time to teach an ever-increasing amount of foundational science, both basic and social, as well as address current topic gaps such as nutrition. By integrating small blocks of time (no more than one hour at a time) into the student’s busy schedule, we successfully designed an effective, efficient learning experience totaling no more than 2.5 hours. The program was well-received and promoted dietary change in a substantial number of students.

This was the first-time nutrition was included as a wellness component in our medical school curriculum. Our model could be adapted to other wellness components such as physical activity and sleep. Each of these could have an active, hands-on learning component, for example, tracking steps for physical activity, followed with a traditional didactic presentation, an interactive large group discussion, and a three-month follow-up evaluation.

Food data can be collected in a variety of different ways, including food history, food frequency, and dietary recall. Each provides a slightly different type of data. Food recall was selected to provide the student with a snapshot of their current dietary intake since they had been enrolled in school for three weeks and had begun to develop their school habits. We wanted to do this without adding an excessive time burden to the students already crowded curricular schedule. Students were asked to complete three 24-hour recalls, one weekend day and two weekdays, because a one-day recall would not adequately represent the student’s nutrient and food group intake. Despite a few students commenting that the process was time-consuming, three-fourths of the students completed three or more recalls.

Many diet tracking or food recall programs are freely available on the web or mobile devices. For this experience, it was essential to pick a diet tracking tool that provided both food group and nutrient information so the students could connect nutrients with specific foods, such as vitamin C with fruits and vegetables. Also, the inclusion of food groups allowed us to demonstrate MyPlate, a quick diet assessment tool that uses food groups and can easily be used anywhere, such as in the clinic, and without additional teaching materials. At the time of this experience, the ASA24 was the only free diet tracking program available that provided food group information. The ASA24 program permitted students to identify nutrients or food groups in their personal diets that met or exceeded the recommended intake, and to compare the group’s intake to these dietary recommendations. This program is an empirically based, validated diet tracking program [37,38] and provides a more extensive list of nutrients, unlike most of the available free diet tracking programs or apps.

Diet tracking methods, such as food recalls, have several limitations. Some are unique to the method, such as access to a camera if the diet tracking method uses pictures to record what was eaten. Food recalls rely on recall of what was consumed the day before and often underreport the amount of food eaten. Studies have shown that the ASA24 collects information on foods consumed as reliably as an interviewer-administered 24-hour recall [39]. For this reason, we used the food recall as the hands-on learning activity to give the students an overview of their nutrient and food group intake so they could make changes to their diet and improve its quality.

Since the ASA24 does not collect personal health information, the student’s energy requirements could not be calculated and compared to their actual caloric intake. During the presentation, the class’s overall energy intake was given, but it was not compared to a recommended value like the 2000 calories associated with the Daily Value (DV) and the nutrition facts panel on package labels. We avoided the comparison because the 2000 calories may be over or under some students’ caloric needs. For example, if a student were physically active, then the 2000 calories would underestimate their calorie need. If the student was short in stature and underweight, then the 2000 calories may overestimate their need.

Several nutrients did not meet the recommended Daily Value (DV). A few studies have examined the nutrient intake of medical students, but none have reported intake upon entering medical school. Bergeron et al. [18] assessed the dietary practices of pharmacy and medical students in California using a food frequency questionnaire. They reported that a large percentage of students did not meet many of the dietary recommendations; for example, only 13% met fiber intake goals, and 10% consumed the recommended amount of fruit and vegetables. Brehm et al. followed a cohort of 125 medical students for four years and reported most micronutrient intakes met the DRI except for an inadequate intake of vitamin D and an excess of sodium [7,40]. Fredrikson et al. [41] collected 3-day food records from 698 Swedish medical students and found vitamin D intake was below the recommended level. Sodium intake was not quantified. Our data support the findings of both these studies in that. Sodium intake was excessive, and vitamin D intake was below the DRI.

The Healthy Eating Index [25,42], developed by the USDA, is a measure of diet quality [43]. The score ranges from 0 to 100, and a score of 100 means the individual’s food intake aligns with key dietary recommendations from the 2015–2020 *Dietary Guidelines for Americans* [43]. Diets that score high on the HEI are associated with a significant reduction in the risk of all-cause mortality, cardiovascular disease, cancer, type 2 diabetes, and neurodegenerative disease [44]. The students that participated in this experience (Table 2) had an HEI score of 59, which is the same for Americans overall [45]. A score that falls short of the Dietary Guidelines and is no better than the general population speaks to the importance of including nutrition education in the medical school curriculum, both for current student well-being and future clinical practices.

The ASA24 also reports several non-nutrient substances like caffeine, added sugar, and alcohol. Our group consumed caffeine primarily in the form of coffee and most consumed an amount of caffeine similar to one cup of coffee per day (95 mg/8 oz) [46], consistent with that observed for college students. However, another recent study of college students (n = 1248) found 92% of the students consumed caffeine in any form and that coffee was the primary source. In this study, college students consumed approximately 20% more caffeine [47] than our students. For added sugar, the recommended DV is 50 g per day or less [21], and the average American adult consumes more than 100 g per day [48]. Surprisingly, our group’s added sugar intake was significantly lower, reported at only 10 g per day.

Fewer than 1 in 5 of the students reported alcohol intake on at least one recall. It is unknown if the alcohol was consumed as a beverage or used in cooking, but it is assumed that most consumed the alcohol as a beverage since the average intake was 2 drinks per occasion. Most consumed alcohol in moderation, assuming the male level since sex was not known. There were 4 students whose intake was greater than two drinks a day. Still, one must be careful not to draw inferences since self-reporting of alcohol intake is not a reliable indicator of one’s chronic alcohol use. There is growing concern over excessive use of alcohol by medical students, and a national study found one in 3 American medical students met the criteria for alcohol abuse [49].

Our students recommended that future classes discuss food selection for individuals with limited time and funds. Vilaro et al. [50] also reported time (convenience) and cost as predictors of food choices among college students. In college students, a higher price and/or a busier daily life predicted a lower fruit and vegetable intake. Our student’s fruit and vegetable intake were below the MyPlate recommendation for a 2000 kcal diet, but we do not know whether cost or time affected their food choices.

Our students also made several other recommendations that we are incorporating into subsequent years. One suggestion was to modify MyPlate from a PowerPoint presentation to a more interactive session. One way to do that would be to have the students use the MyPlate website [19] to develop their plan and then discuss in small teams whether their own diets fell short of the MyPlate recommendations. In 2020, due to COVID-19 restrictions, we had students form small groups virtually and brainstorm ideas on how to bring their diet up to MyPlate recommendations.

## Conclusion

In conclusion, we describe a brief, interactive nutrition experience designed to increase incoming medical students’ knowledge of their own dietary behavior and to improve their nutritional health. Another goal was to heighten their nutrition awareness to make them better nutrition advocates with their patients. This experience resulted in some students making changes to their diet to fill their nutrition gaps. The students also recommended the program be continued in future years.

## References

[1] Dyrbye LN, West CP, Satele D, et al. Burnout among U.S. medical students, residents, and early career physicians relative to the general U.S. population. Acad Med. 2014;89(3):443–12. PubMed PMID: 24448053. 24448053 10.1097/ACM.0000000000000134

[2] Drolet BC, Rodgers S. A comprehensive medical student wellness program–design and implementation at Vanderbilt School of Medicine. PubMed PMID: 20042835 Acad Med. 2010;85 1:103–110. 20042835 10.1097/ACM.0b013e3181c46963

[3] Curricular Initiatives n.d. Available from: http://med.stanford.edu/md/student-affairs/student-wellness/curriculum.html

[4] Green, S. *Food as medicine*: *Integrating nutrition education into the Medical Education Curriculum*. 2018. Cited [Aug 1, 2019) Available from: https://geiselmed.dartmouth.edu/news/2018/food-as-medicine-integrating-nutrition-education-into-the-medical-education-curriculum/

[5] Frank E, Carrera JS, Elon L, et al. Basic demographics, health practices, and health status of U.S. medical students. Am J Prev Med. 2006;31(6):499–505. Epub 2006/ 12/16. doi: 10.1016/j.amepre.2006.08.009. PubMed PMID: 17169711. 17169711

[6] Frank E, Wright EH, Serdula MK, et al. Personal and professional nutrition-related practices of US female physicians. Am J Clin Nutr. 2002;75(2):326–332. Epub 2002/ 01/30. doi: 10.1093/ajcn/75.2.326. PubMed PMID: 11815326. 11815326

[7] Brehm BJ, Summer SS, Khoury JC, et al. Health status and lifestyle habits of US medical students: A longitudinal study. Ann Med Health Sci Res. 2016;6(6):341–347. Epub 2017/05/26. doi: 10.4103/amhsr.amhsr_469_15. PubMed PMID: 28540101; PubMed Central PMCID: PMCPMC5423333. 28540101 PMC5423333

[8] MacLean L, Booza J, Balon R. The impact of medical school on student mental health. Acad Psychiatry. 2016;40(1):89–91. Epub 2015/03/10. doi: 10.1007/s40596-015-0301-5. PubMed PMID: 25749920. 25749920

[9] Majra J. Do our medical colleges inculcate health-promoting lifestyle among medical students: a pilot study from two medical colleges from southern India. Int J Prev Med. 2013 Epub 2013/05/15. PubMed PMID: 23671774; PubMed Central PMCID: PMCPMC3650594;4(4):425–429. . 23671774 PMC3650594

[10] Kjeldstadli K, Tyssen R, Finset A, et al. Life satisfaction and resilience in medical school–a six-year longitudinal, nationwide and comparative study. BMC Med Educ. 2006;6:48. Epub 2006/ 09/21. PubMed PMID: 16984638; PubMed Central PMCID: PMCPMC1592096. . 16984638 10.1186/1472-6920-6-48 PMC1592096

[11] Frank E, Carrera JS, Elon L, et al. Predictors of US medical students’ prevention counseling practices. Prev Med. 2007;44(1):76–81. Epub 2006/ 09/19. doi: 10.1016/j.ypmed.2006.07.018. PubMed PMID: 16978687. 16978687

[12] Km Bw A, Kohlmeier M. The state of nutrition education at US medical schools. J Biomed Educ. 2015. DOI:10.1155/2015/357627.

[13] Horrocks PM, Blackmore R, Wright AD. A long-term follow-up of dietary advice in maturity onset diabetes: the experience of one centre in the UK prospective study. Diabet Med. 1987;4(3):241–244. Epub 1987/05/01. PubMed PMID: 2956027. 2956027 10.1111/j.1464-5491.1987.tb00871.x

[14] Ammerman AS, DeVellis RF, Carey TS, et al. Physician-based diet counseling for cholesterol reduction: current practices, determinants, and strategies for improvement. Prev Med. 1993;22(1):96–109. Epub 1993/ 01/01. . PubMed PMID: 8475015. 8475015 10.1006/pmed.1993.1007

[15] Willcox DC, Willcox BJ, Todoriki H, et al. The Okinawan diet: health implications of a low-calorie, nutrient-dense, antioxidant-rich dietary pattern low in glycemic load. PubMed PMID: 20234038 J Am Coll Nutr. 2009;28 Suppl:500S–16S. 20234038 10.1080/07315724.2009.10718117

[16] Shao ADA, Willcox DC, Kramer L, et al. Optimal nutrition and the ever-changing dietary landscape: a conference report. Eur J Nutr. 2017;56(Suppl 1):S1–S21. . 10.1007/s00394-017-1460-9 PMC5442251 28474121

[17] Singh, K. Nutrient and Stress Management. J Nutr Food Sci. 2016;6(4): 528.

[18] Bergeron N, Al-Saiegh S, Ip EJ. An analysis of California pharmacy and medical students’ dietary and lifestyle practices. PubMed PMID: 29200450; PubMed Central PMCID: PMCPMC5701325 Am J Pharm Educ. 2017;81 8:5956. 29200450 10.5688/ajpe5956 PMC5701325

[19] Subar AF, Kirkpatrick SI, Mittl B, et al. The automated self-administered 24-hour dietary recall (ASA24): a resource for researchers, clinicians, and educators from the national cancer institute. J Acad Nutr Diet. 2012;112(8):1134–1137. Epub 2012/ 06/19. doi: 10.1016/j.jand.2012.04.016. PubMed PMID: 22704899; PubMed Central PMCID: PMCPMC3721511. 22704899 PMC3721511

[20] U.S. Department of Agriculture. ChooseMyPlate.gov Washington, DC. [cited Aug 1, 2019] Available from: https://www.choosemyplate.gov

[21] FDA. Daily value and percent daily value: changes on the new nutrition and supplement facts labels in: FDA, editor. 2020.

[22] USDA. MyPlate Plan. Available from: https://www.choosemyplate.gov/resources/MyPlatePlan.

[23] US Food and Drug Administration. How to understand and use the nutrition facts label Washington D.C.2019 [cited 201982, 2019]. Available from: https://www.fda.gov/food/new-nutrition-facts-label/how-understand-and-use-nutrition-facts-label.

[24] US Food and Drug Administration. Added sugars on the new nutrition facts label Washington DC2020 [cited 202081, 2019]. Available from: https://www.fda.gov/food/new-nutrition-facts-label/added-sugars-new-nutrition-facts-label#:~:text=They%20do%20not%20include%20naturally,goods%2C%20desserts%2C%20and%20sweets.

[25] Heimburger DC, Ullmann DO, Ramsey MJ, et al. Dietary habits of first-year medical students assessed during clinical nutrition course. Nutrition. 1994;10(3):214–220. Epub 1994/ 05/01. PubMed PMID: 7919672. 7919672

[26] Epidemiology and Genomics Research Program. SAS code Washington DC. 2015 [cited 201981, 2019]. Available from: https://epi.grants.cancer.gov/hei/sas-code.html.

[27] Shea S, Basch CE, Zybert P. Correlates of internists’ practices in caring for patients with elevated serum cholesterol. Am J Health Promot. 1990;4(6):421–428. Epub 1990/ 07/01. doi: 10.4278/0890-1171-4. PubMed PMID: 22204620. 22204620

[28] Hyman DJ, Maibach EW, Flora JA, et al. Cholesterol treatment practices of primary care physicians. 433021. 1992 Epub 1992/ 07/01. PubMed PMID: 1641441; PubMed Central PMCID: PMCPMC1403675;107(4):441–448. . PMC1403675 1641441

[29] Levine BS, Wigren MM, Chapman DS, et al. A national survey of attitudes and practices of primary-care physicians relating to nutrition: strategies for enhancing the use of clinical nutrition in medical practice. Am J Clin Nutr. 1993;57(2):115–119. Epub 1993/ 02/01. doi: 10.1093/ajcn/57.2.115. PubMed PMID: 8424377. 8424377

[30] Russell NK, Roter DL. Health promotion counseling of chronic-disease patients during primary care visits. Am J Public Health. 1993;83(7):979–982. Epub 1993/ 07/01. doi: 10.1093/ajcn/57.2.115. PubMed PMID: 8328620; PubMed Central PMCID: PMCPMC1694773. 8328620 PMC1694773

[31] Kushner RF. Barriers to providing nutrition counseling by physicians: a survey of primary care practitioners. Prev Med. 1995;24(6):546–552. Epub 1995/ 11/01. doi: 10.1006/pmed.1995.1087. PubMed PMID: 8610076. 8610076

[32] Wechsler H, Levine S, Idelson RK, et al. The physician’s role in health promotion revisited–a survey of primary care practitioners. N Engl J Med. 1996;334(15):996–998. Epub 1996/ 04/11. doi: 10.1056/NEJM199604113341519. PubMed PMID: 8596615. 8596615

[33] Sciamanna CN, DePue JD, Goldstein MG, et al. Nutrition counseling in the promoting cancer prevention in primary care study. Prev Med. 2002;35(5):437–446. Epub 2002/ 11/15. doi: 10.1006/pmed.2002.1099. PubMed PMID: 12431892. 12431892

[34] Anis NA, Lee RE, Ellerbeck EF, et al. Direct observation of physician counseling on dietary habits and exercise: patient, physician, and office correlates. Prev Med. 2004;38(2):198–202. Epub 2004/ 01/13. doi: 10.1016/j.ypmed.2003.09.046. PubMed PMID: 14715212. 14715212

[35] Ramsetty A, Adams C, Berini C, et al. Medical student attitudes on nutrition counseling after implementation of a novel curricular activity. J Am Coll Nutr. 2020;39(4):333–337. Epub 2019/ 09/14. . PubMed PMID: 31518212. 31518212 10.1080/07315724.2019.1659191

[36] Taylor GW, Stumpos ML, Kerschbaum W, et al. Educating dental students about diet-related behavior change: does experiential learning work? J Dent Educ. Epub 2014/ 01/05. PubMed PMID: 24385526. 2014;78(1):64–74. 24385526

[37] Kirkpatrick SI, Subar AF, Douglass D, et al. Performance of the automated self-administered 24-hour recall relative to a measure of true intakes and to an interviewer-administered 24-h recall. Am J Clin Nutr. 2014;100(1):233–240. Epub 2014/ 05/03. doi: 10.3945/ajcn.114.083238. PubMed PMID: 24787491; PubMed Central PMCID: PMCPMC4144101. 24787491 PMC4144101

[38] Timon CM, Van Den Barg R, Blain RJ, et al. A review of the design and validation of web- and computer-based 24-h dietary recall tools. Nutr Res Rev. 2016;29(2):268–280. Epub 2016/ 12/14. doi: 10.3945/ajcn.114.083238. PubMed PMID: 27955721. 27955721

[39] Thompson FE, Dixit-Joshi S, Potischman N, et al. Comparison of interviewer-administered and automated self-administered 24-hour dietary recalls in 3 diverse integrated health systems. Am J Epidemiol. 2015;181(12):970–978. Epub 2015/ 05/13. doi: 10.1093/aje/kwu467. PubMed PMID: 25964261; PubMed Central PMCID: PMCPMC4462333. 25964261 PMC4462333

[40] Hidiroglu S, Tanriover O, Unaldi S, et al. A survey of energy-drink consumption among medical students. J Pak Med Assoc. 2013 Epub 2013/ 08/02. PubMed PMID: 23901705;63(7):842–845. . 23901705

[41] Fredriksson E, Brekke HK, Ellegard L. Dietary intake in Swedish medical students during 2007-2012. Scand J Public Health. 2016;44(1):77–83. Epub 2015/ 10/22. doi: 10.1177/1403494815611767. PubMed PMID: 26487764. 26487764

[42] USDA Department of Agriculture. Healthy eating index 2019 [cited 201981, 2019]. Available from: https://www.fns.usda.gov/resource/healthy-eating-index-hei.

[43] USDA Department of Agriculture. 2015 – 2020 Dietary guidelines for Americans. Washington, DC. December 2015. 8th. [cited Aug 1, 2019]. Available from: https://health.gov/our-work/food-and-nutrition/2015-2020-dietary-guidelines/

[44] Schwingshackl L, Bogensberger B, Hoffmann G. Diet quality as assessed by the healthy eating index, alternate healthy eating index, dietary approaches to stop hypertension score, and health outcomes: an updated systematic review and meta-analysis of cohort studies. J Acad Nutr Diet. 2018;118(1):74–100e11. Epub 2017/ 11/08. doi: 10.1016/j.jand.2017.08.024. PubMed PMID: 29111090. 29111090

[45] National Center for Health Statistics. What we eat in America/national health and nutrition examination survey, 2013-2014. Healthy Eating Index-2015 [cited 201981 2019].

[46] U.S. Department of Agriculture, Agricultural Research Service. Food Data Central, 2019. Available from: https://fdc.nal.usda.gov.

[47] Mahoney CR, Giles GE, Marriott BP, et al. Intake of caffeine from all sources and reasons for use by college students. Clin Nutr. 2019;38(2):668–675. Epub 2018/04/24. doi: 10.1016/j.clnu.2018.04.004 PubMed PMID: 29680166. 29680166

[48] Newens KJ, Walton J. A review of sugar consumption from nationally representative dietary surveys across the world. J Hum Nutr Diet. 2016;29(2):225–240. Epub 2015/10/11. doi: 10.1111/jhn.12338 PubMed PMID: 26453428; PubMed Central PMCID: PMCPMC5057348. 26453428 PMC5057348

[49] Jackson ER, Shanafelt TD, Hasan O, et al. Burnout and alcohol abuse/dependence among U.S. medical students. Acad Med. 2016;91(9):1251–1256. Epub 2016/03/05. doi: 10.1097/ACM.0000000000001138 PubMed PMID: 26934693. 26934693

[50] Vilaro MJ, Colby SE, Riggsbee K, et al. Food choice priorities change over time and predict dietary intake at the end of the first year of college among students in the U.S. Nutrients. 2018;10(9) Epub 2018/09/16. 10.3390/nu10091296. PubMed PMID: 30217004; PubMed Central PMCID: PMCPMC6164337. PMC6164337 30217004

